# Real-Time Scanning Ion Conductance Microscopy Reveals MARCKS-Driven Morphological Remodeling and Nanomechanical Softening during LPS-Induced Macrophage Polarization

**DOI:** 10.1021/acs.analchem.6c01115

**Published:** 2026-08-05

**Authors:** Dong Wang, Ritsuko Yamase, Kyunghee Park, Hiromi Nishikawa, Andrew Shevchuk, Rebecca C. Coll, Yuri Korchev, Wei Wang, Yanjun Zhang

**Affiliations:** † WPI Nano Life Science Institute (WPI-NanoLSI), 12858 Kanazawa University, Kanazawa 920-1192, Japan; ‡ Department of Metabolism, Digestion and Reproduction, 4615 Imperial College London, London W12 0NN, U.K.; § Wellcome-Wolfson Institute for Experimental Medicine, Queen’s University Belfast, Belfast BT9 7BL, U.K.

## Abstract

Macrophages initiate acute immune responses to danger signals through highly plastic polarization and dynamic remodeling of cell mechanics and morphology. While physical cues are increasingly recognized as coregulators of macrophage activation alongside biochemical signals, the role of pathogen-induced biophysical remodeling in shaping immune function remains poorly understood. In this study, we employed noncontact scanning ion conductance microscopy (SICM) to monitor real-time nanomechanical and morphological changes in immortalized bone marrow-derived macrophages (iBMDMs) at single-cell resolution during lipopolysaccharide (LPS)-induced M1 polarization. We found that M1 activation significantly increased the surface area-to-volume (SA/V) ratio and decreased cellular stiffness—biophysical adaptations that may support enhanced pro-inflammatory responses. Mechanistically, these changes were driven by NF-κB–dependent activation of the cytoskeletal regulator MARCKS. The inhibition of MARCKS disrupted both morphological remodeling and inflammatory activation, highlighting its essential role in coordinating immune signaling with nanomechanical plasticity. This study provides the first real-time, quantitative evidence linking inflammatory signaling to macrophage morphological and nanomechanical remodeling, and shows that MARCKS inhibition suppresses, while PMA-mediated activation mimics, LPS-induced responses.

## Graphical abstract

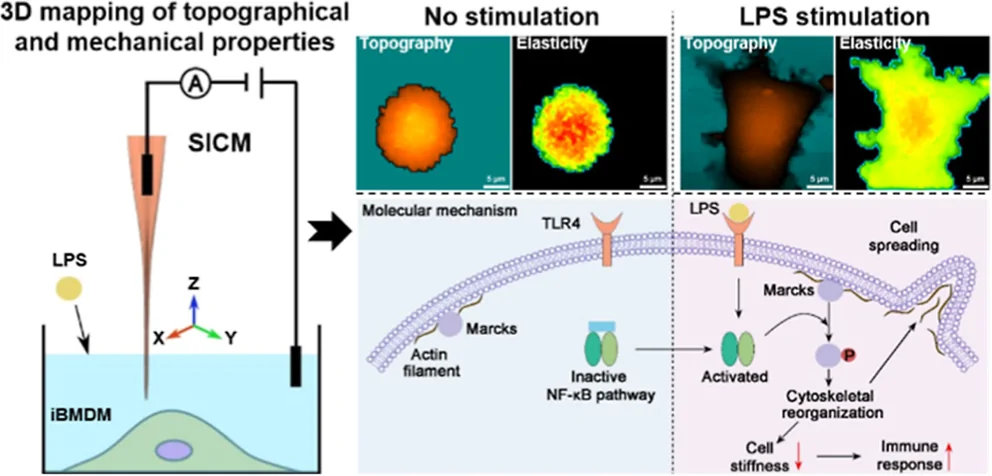

## Introduction

Macrophages are central players in the acute immune response, mediating pathogen and cancer cell clearance, apoptotic cell removal, and antigen presentation.^1,2^ Their functions depend on high polarization plasticity into classically activated M1 (pro-inflammatory) or alternatively activated M2 (anti-inflammatory) states in response to dynamic microenvironmental signals. M1 macrophage polarization is classically induced by lipopolysaccharide (LPS), a Toll-like receptor 4 (TLR4) agonist, in combination with pro-inflammatory cytokines such as IFN-γ and TNF-α, whereas M2 polarization is primarily driven by IL-4 and IL-13, and can also be promoted by additional signals including IL-10, TGF-β, and glucocorticoids.^3,4^ Beyond biochemical signals, physical factors such as tensile stress and hydrostatic pressure—especially in swollen, inflamed tissues—also coregulate macrophage plasticity and morphology.^4–6^ A soft extracellular matrix (ECM) promotes M1 polarization with a round, spreading morphology, whereas a stiff ECM favors M2 polarization with an elongated shape.^7,8^ In addition to external mechanical cues, the intrinsic mechanical properties of macrophages are also linked to their polarization states.^4,9^ Although M1 macrophages are essential for host defense, the signaling mechanisms regulating their activation—and the relationship between nanomechanical properties and morphological remodeling during M1 polarization—remain incompletely understood, highlighting the need for real-time monitoring of these dynamic changes.^1^

Atomic force microscopy (AFM), a widely used scanning probe microscopy (SPM) technique, has been applied to characterize macrophage morphology and mechanics during M1 activation.^10–12^ However, compared with M0, some studies report that M1 macrophages exhibit a spread morphology with increased stiffness, while others suggest they are mechanically softer.^11,13^ Despite this variability, mechanical properties—whether stiffer or softer—consistently correlate with macrophage phenotype during M0-to-M1 polarization. Real-time monitoring of the same individual macrophages before and after stimulation is essential to accurately capture relative morphological and nanomechanical changes and to elucidate the underlying mechanisms. However, the high plasticity, sensitivity, and rapid morphological responses of macrophages pose significant challenges for long-term, repetitive AFM scanning.^14,15^

Recently, noncontact scanning ion conductance microscopy (SICM) has emerged as a powerful alternative for real-time analysis of cell morphology and nanomechanics.^16–18^ Unlike AFM, which deforms the cell surface and limits long-term imaging,^14,15^ SICM enables repeated, noninvasive measurements of the same cell over time without structural damage or unintended stimulation.^15,17,18^ While the surface area-to-volume (SA/V) ratio is a key morphological parameter influencing cellular function and interactions with the environment,^19,20^ most existing methods measure only 2D surface area and overlook dynamic 3D volume changes during macrophage activation.^21,22^ In contrast, SICM enables simultaneous high-resolution 3D morphology, nanomechanical mapping, as well as quantification of both surface area and volume in a single scan,^23^ making it well suited for studying LPS-induced morphological and mechanical changes in macrophages.

Cytoskeletal remodeling is essential for rapid macrophage morphological and nanomechanical changes, enabling acute immune responses such as migration, pathogen phagocytosis, and antigen presentation.^4,24^ MARCKS, a membrane-associated actin-binding protein highly expressed in macrophages, regulates cytoskeletal dynamics and is implicated in controlling macrophage morphology, nanomechanical properties, and LPS-induced immune activation.^25,26^ However, the precise role of MARCKS in macrophage function remains unclear.^26^ Here, we used SICM to monitor real-time 3D morphological and nanomechanical remodeling during LPS-induced M1 polarization in immortalized bone marrow-derived macrophages (iBMDMs). LPS stimulation induced cell spreading, characterized by an increased SA/V ratio and reduced stiffness, driven by NF-κB-dependent activation of the cytoskeletal regulator MARCKS. These biophysical changes enhanced pro-inflammatory responses, revealing a MARCKS-dependent mechanobiological pathway that links inflammatory signaling to macrophage function. This study provides the first real-time quantification of coordinated 3D morphological and nanomechanical remodeling during macrophage activation and establishes SICM as a powerful, label-free tool for single-cell immune response analysis.

## Materials and Methods

### Immortal Bone-Marrow-Derived Macrophages (iBMDM) Culture, LPS Stimulation, and Chemical Inhibitors

The immortalized bone marrow-derived macrophage (iBMDM) cell line derived from wild-type mice (BEI Resources, NR-9456) is cataloged in the Cellosaurus database as BoMaMa-J2imm (accession: CVCL_A7WX) and can be referenced using the Research Resource Identifier (RRID:CVCL_A7WX). Immortalized bone marrow-derived macrophages (iBMDMs) were cultured in Dulbecco’s Modified Eagle’s Medium (DMEM; Invitrogen, Carlsbad, CA, USA) supplemented with 10% fetal calf serum (FCS), 1% penicillin–streptomycin, and 1% sodium pyruvate (Thermo Fisher Scientific, Waltham, MA, USA). Cells used in this study were maintained between passages 10 and 20. Cells were routinely tested for mycoplasma contamination using ABM’s Mycoplasma PCR Detection Kit (Cat. No. G238) and were confirmed to be mycoplasma negative.

Cells were stimulated with 1 μg/mL lipopolysaccharide (LPS; L2630, Sigma-Aldrich, St. Louis, MO, USA) for either 6 or 16 h, unless otherwise specified. For real-time SICM scanning experiments, LPS stimulation was applied as indicated.

The compounds used in this study—including Jasplakinolide (JAS; actin polymerization inducer, 30 nM), BAY 11-7082 (NF-κB inhibitor, 3 μM), and BIO-11006 (MARCKS inhibitor, 50 μM), all purchased from MedChemExpress—were preincubated with cells for 30 min, followed by LPS stimulation for the indicated durations. Phorbol 12-myristate 13-acetate (PMA; a MARCKS activator) was purchased from Sigma-Aldrich (P8139) and applied to cells at concentrations of 100 and 500 nM for 3 h.

### Fabrication of the SICM Scanning Glass Nanopipettes

To fabricate nanopipettes for scanning ion conductance microscopy (SICM), borosilicate glass capillaries (ID: 0.58 mm; OD: 1.00 mm; Harvard Apparatus, UK) were pulled using a CO_2_ laser-based micropipette puller (Model P-2000, Sutter Instruments Co., USA), following a previously established two-step protocol.^18^ In the first stage, parameters were set to heat 350, filament 3, velocity 30, and delay 200. The second stage involved: heat 350, filament 2, velocity 27, delay 160, and pull 250. This fabrication procedure yielded SICM nanopipettes with tip diameters of ∼ 100 nm and a half-cone angle (α) of ∼3°, as confirmed by scanning electron microscopy (Figure S9). Before each SICM scanning, the nanopipettes were routinely checked by their electrical resistance, which was ∼100 MΩ when filled with phosphate-buffered saline (PBS).

### SICM Operating in Hopping Probe Mode and Nanomechanical Properties Measuring Protocol

The iBMDM cells were seeded at a density of 1 × 10^4^ cells/cm^2^ into 35 mm Petri-dish. After 2 days of culture in a CO_2_ incubator, the medium was replaced with CO_2_-independent medium (Gibco, Thermo Fisher Scientific) for morphology and mechanical property investigations using SICM at room temperature. SICM imaging and nanomechanical property measurements were performed in adaptive resolution hopping probe mode.^16,18^ The system was equipped with a P-730.4C XY nanopositioner (40 × 40 μm) and a P-753.21C *Z*-axis piezo actuator (25 μm travel) (Physik Instrumente, Germany), mounted on a Nikon Eclipse Ti-2 inverted microscope and enclosed in a Faraday cage. Nanopipette control and data acquisition were handled by an ICAPPIC controller and custom software (ICAPPIC Ltd., UK). Ion current was amplified using a MultiClamp 700B (Molecular Devices, USA) with a 2 kHz low-pass filter, digitized via Digidata 1550B, and recorded using Clampex 10.7. A holding potential of +200 mV was applied during scans. During SICM imaging and mechanical mapping, the nanopipette approached the cell surface at 200 μm s^–1^. Topographic images were acquired at 256 × 256 pixel resolution using adaptive scanning mode with an ∼11 μm hopping amplitude to accommodate cell height and avoid probe–sample contact. A typical 30 × 30 μm^2^ scan required approximately 9 min. A three-set point imaging protocol was used with ion current reductions of 0.5%, 1%, and 2% relative to the reference current measured far from the surface. The 0.5% set point provided near-noncontact topographic images for 3D reconstruction and calculation of cellular surface area and volume. Images acquired at 1% and 2% set points involved progressively smaller tip–sample distances, increasing hydrodynamic interaction and inducing controlled macrophage deformation. Local deformation was determined from the apparent height difference between the 1% and 2% set point images.

The intrinsic force *F* between the nanopipette and the sample was calibrated using a decane droplet model validated in our previous work,^17^ governed by interfacial tension at the decane–saline interface and estimated as

$$F=4\pi \sigma \sqrt{Rd}$$

where σ is the surface tension, *R* is the nanopipette radius, and *d* is the maximum indentation depth. In this study, σ = 0.25 × 10^–3^ N/m was used.^27^ For nanopipettes with *R* ≈ 50 nm (Figure S9), *d* ≈ 35 nm was obtained from established calibration curves,^17^ yielding an intrinsic force of ∼130 pN. The effective applied pressure was calculated as previously described^28^ using *p* = *F*/*S*, where the effective contact area is defined as *S* = π*Rd*. This yielded an effective pressure of approximately 24 kPa. Young’s modulus was computed using the previously described model,^29^ and local maps *E*(*x*,*y*) were generated using SICM ImageViewer (ICAPPIC Ltd., London, UK) following established procedures.^17^

### Western Blotting

1 × 10^6^ iBMDM cells treated with LPS for 6 h in the presence or absence of 30 min pretreatment of compounds (BIO-11006, BAY 11-7082) were lysed using RIPA (20 mM Tris–HCl pH 7.4, 150 mM NaCl, 1 mM EDTA, 1% Triton X-100, 10% glycerol, 0.1% SDS and 0.5% deoxycholate) buffer supplemented with complete protease inhibitor (Roche cOmplete). Cell lysates were clarified by centrifugation at 10,000*g* for 10 min, followed by the protein concentration quantification by BCA assay and 10 μg of the protein sample was separated using a 10–15% SDS-polyacrylamide gel. As a size marker, 5 μL of Precision Plus ProteinTM Standards (Bio-Rad, #161-0374) protein ladder were loaded on each gel. Samples were separated under denaturing and reducing electrophoretic conditions at 150 V in Tris-glycine SDS running buffer (25 mM Tris base, 192 mM glycine, 0.1% SDS, pH 8.3). Separated proteins were then transferred from the gel to an Immobilon-FL polyvinylidene difluoride (PVDF) membrane at 32 V for 1–1.5 h. Nonspecific antibody binding to the membrane was blocked by incubation of the membrane with 5% Bovine Serum Albumin (BSA) for at least 1 h at RT. Antibodies diluted in 5% BSA were incubated with the blocked membrane overnight at 4 °C. Membranes were washed three times with TBST and then incubated with the secondary antibody for 1 h at RT in 5% BSA. Membranes were then washed twice in TBST and one last time in TBS before being developed on an ECL detection system (GE Healthcare). Antibodies against phospho-MARCKS (Cell Signaling Technology, #8722T), iNos (Millipore, AB5382), phospho- NF-κB-p65 (Cell Signaling Technology, #3033) and total NF-κB p65 (Cell Signaling Technology, #8242) were used as primary antibodies. Anti-GAPDH antibody (FUJIFILM Wako, #016-25523) was used as the loading control.

### Real-Time RT-PCR

Total RNA was extracted from iBMDM cells using ISOGEN (Nippon Gene, Toyama, Japan), reverse-transcribed using the Prime Script RT Reagent Kit (Takara Bio, Kusatsu, Japan), and amplified using Ex*Taq*II SYBR Premix (Takara Bio) on an Mx3000P real-time thermocycler (Agilent Technologies). Relative mRNA levels of IL-6, TNF-α, IL-10, TGF-β1 to GAPDH were calculated. The primer sequences are shown: IL6 (Forward primer (F): CCACTTCACAAGTCGGAGGCTTA; Reverse primer (R): CCAGTTTGGTAGCATCCATCATTTC); TNF-α (F: TATGGCCCAGACCCTCACA; R: GGAGTAGACAAGGTACAACCCATC); IL-10: (F: GACCAGCTGGACAACATACTGCTAA; R: GATAAGGCTTGGCAACCCAAGTAA); TGF-β1 (F: GTGGTGGAGCAACATGTGGAACTCTA; R: TTGGTTCAGCCACTGCCGTA); GAPDH (F: GGCACAGTCAAGGCCGAGAATG; R: ATGGTGGTGAAGACACCAGTA).

### RNA-seq Data and Mass Spectrometry Data Analysis

RNA-seq data was obtained from Gene Expression Omnibus (GSE200210). Differential expression analysis was performed by using iDEP 2.0 with setting FDR as 0.1. The database of actin cytoskeleton related genes was obtained from Mouse Genome Informatics (MGI), Gene Ontology Browser (GO:0015629).

Mass spectrometry raw data was downloaded from ProteomeXchange Consortium (PXD042097). The normalized data was acquired according to the method described in the published paper.^26^

### F-Actin Labeling

Cells were fixed with 4% methanol-free formaldehyde in PBS for 15 min at room temperature, washed three times with PBS, and permeabilized with 0.25% Triton X-100 for 5 min. After washing, cells were blocked with 1% BSA for 20 min and incubated with DyLight 594 phalloidin (1:300, Cell Signaling Technology, #12877) for 15 min in the dark. Samples were washed, mounted with antifade reagent (VECTASHIELD Vibrance, H-1800-10). DAPI was used for nuclear staining. Fluorescent images were obtained using a TCS SP8 confocal microscope (Leica Microsystems, Wetzlar, Germany).

### Scanning Electron Microscopy (SEM) Imaging of SICM Scanning Glass Nanopipettes

SEM imaging was performed using a dual-beam focused ion beam system (Helios G4 CX, Thermo Fisher Scientific). Images were acquired at accelerating voltages of 1, 5, and 15 kV. Additional details of the imaging parameters are provided alongside the corresponding SEM images.

### Statistical Analysis

Data are presented as mean ± standard deviation (SD). Statistical significance was assessed using two-sided unpaired Student’s *t* tests for Figure 1, Figures S1 and S2, and one-way analysis of variance (ANOVA) for Figures 2, 4, and 5, as well as Figures S5–S8. Differences were considered statistically significant at *P* < 0.05. All analyses were performed using OriginPro 2022 software.

**Figure 1:**
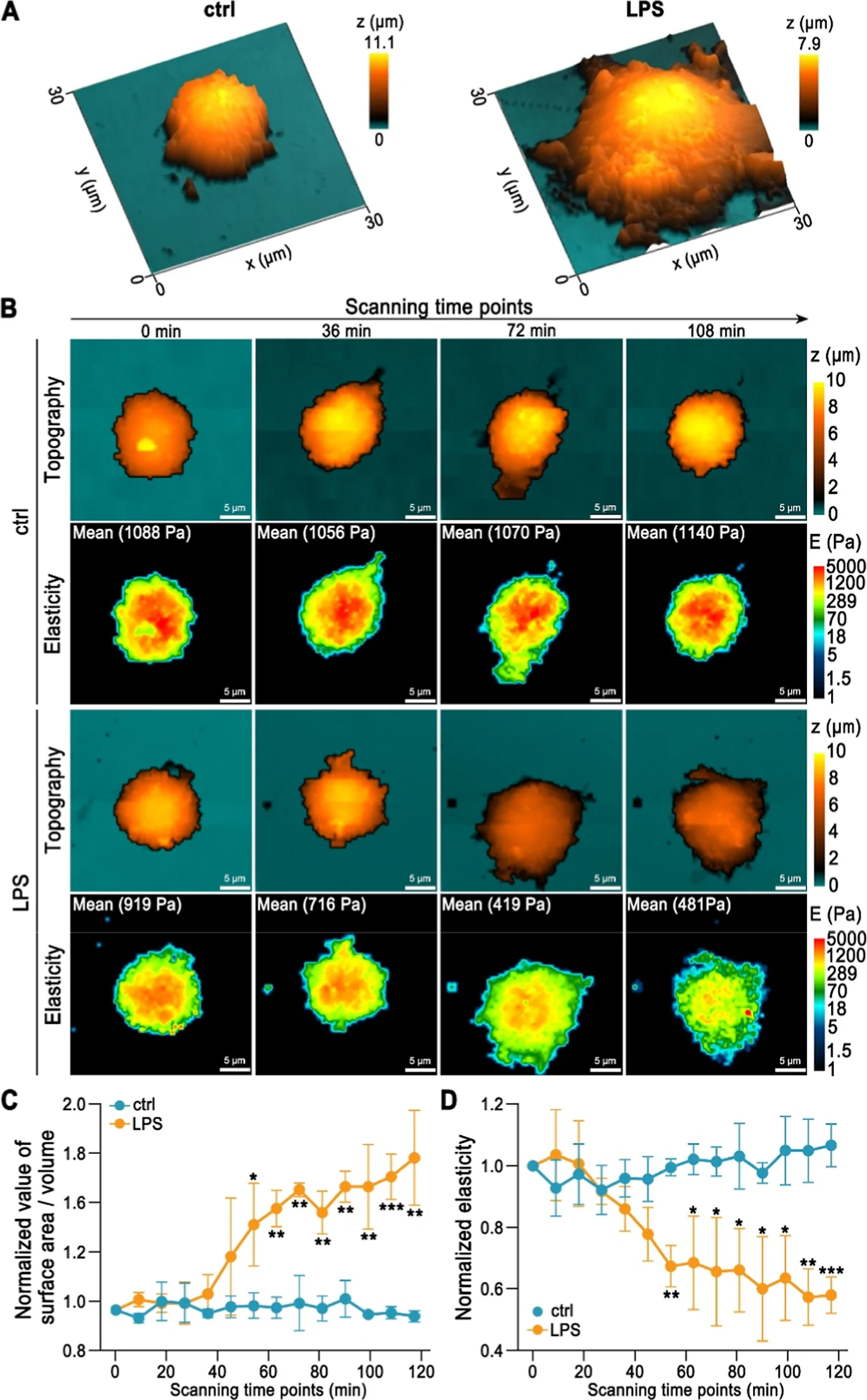
LPS treatment increases surface area-to-volume ratio and decreases elasticity in iBMDMs. (A) Representative 3D SICM images of untreated (ctrl, left) and LPS-treated (right, overnight treatment) iBMDM cells. (B) SICM images showing time-dependent changes in morphology and elasticity of iBMDMs under control and LPS treatment. The selected cell is representative of those analyzed in (C) and (D). Full dynamics are shown in Videos S1 and S2. Changes in SA/V ratio (C) and elasticity (D) of iBMDM cells over time under LPS treatment and control. Each dot represents the mean of three independent experimental repeats. *p < 0.05, **p < 0.01, ***p < 0.001.

**Figure 2:**
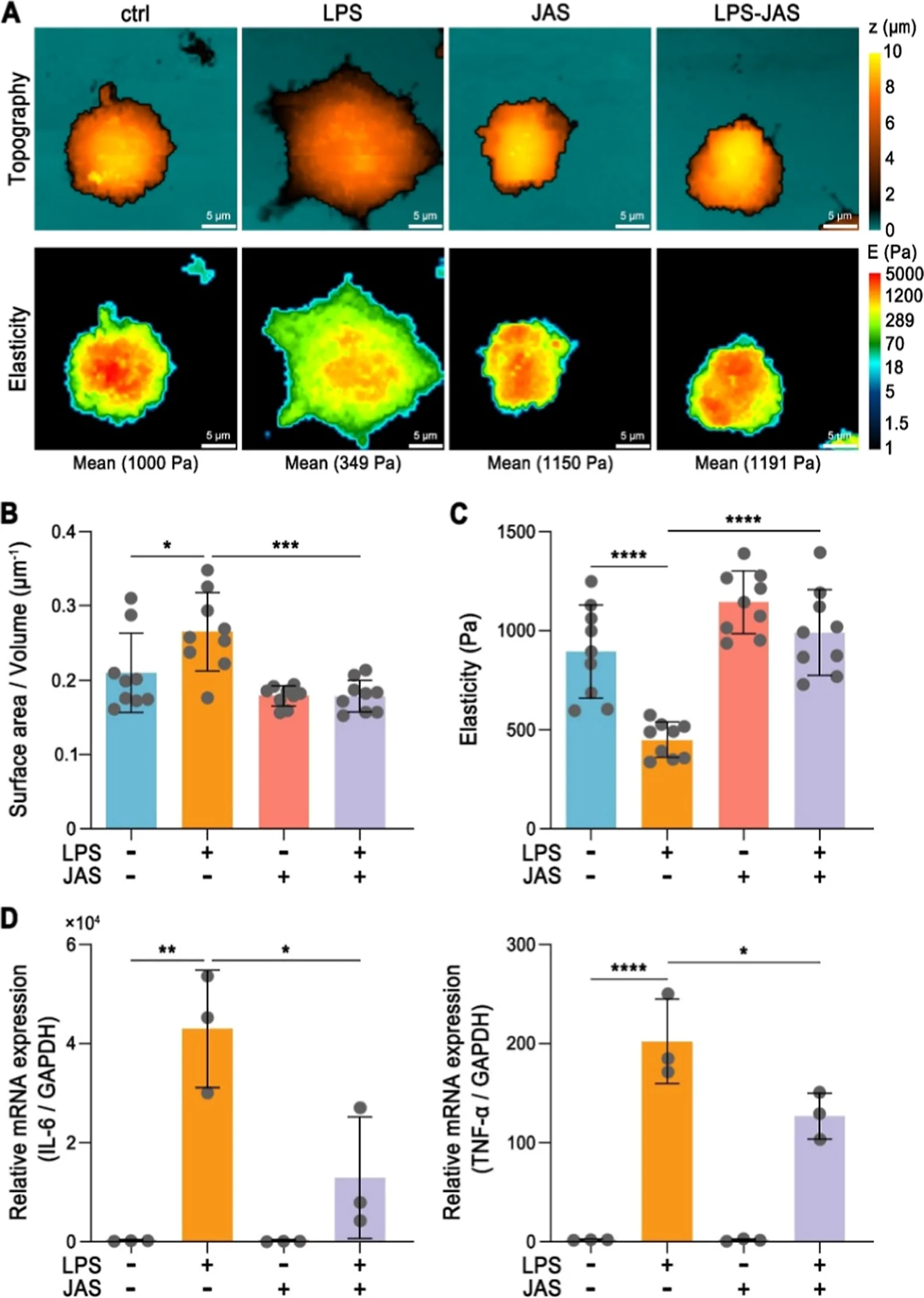
Inhibition of LPS-induced morphological, nanomechanical, and immune changes by Jasplakinolide (JAS). (A) Representative SICM images showing topography (upper) and elasticity (lower) of iBMDM cells under different conditions. Elasticity values are indicated below each image, with height and elasticity represented by the color scale on the right. Bars = 5 μm. (B) SA/V ratio of iBMDMs with different treatments. (C) Elasticity values of iBMDMs with different treatments. Each dot in B and C represents the value from an individual cell, pooled across three independent experimental repeats, *n* = 9 in each group. (D) mRNA expression levels of IL-6 (left) and TNF-α (right) in iBMDMs under various conditions. Data are from three independent experimental repeats. *p < 0.05, **p < 0.01, ***p < 0.001, ****p < 0.0001.

**Figure 3:**
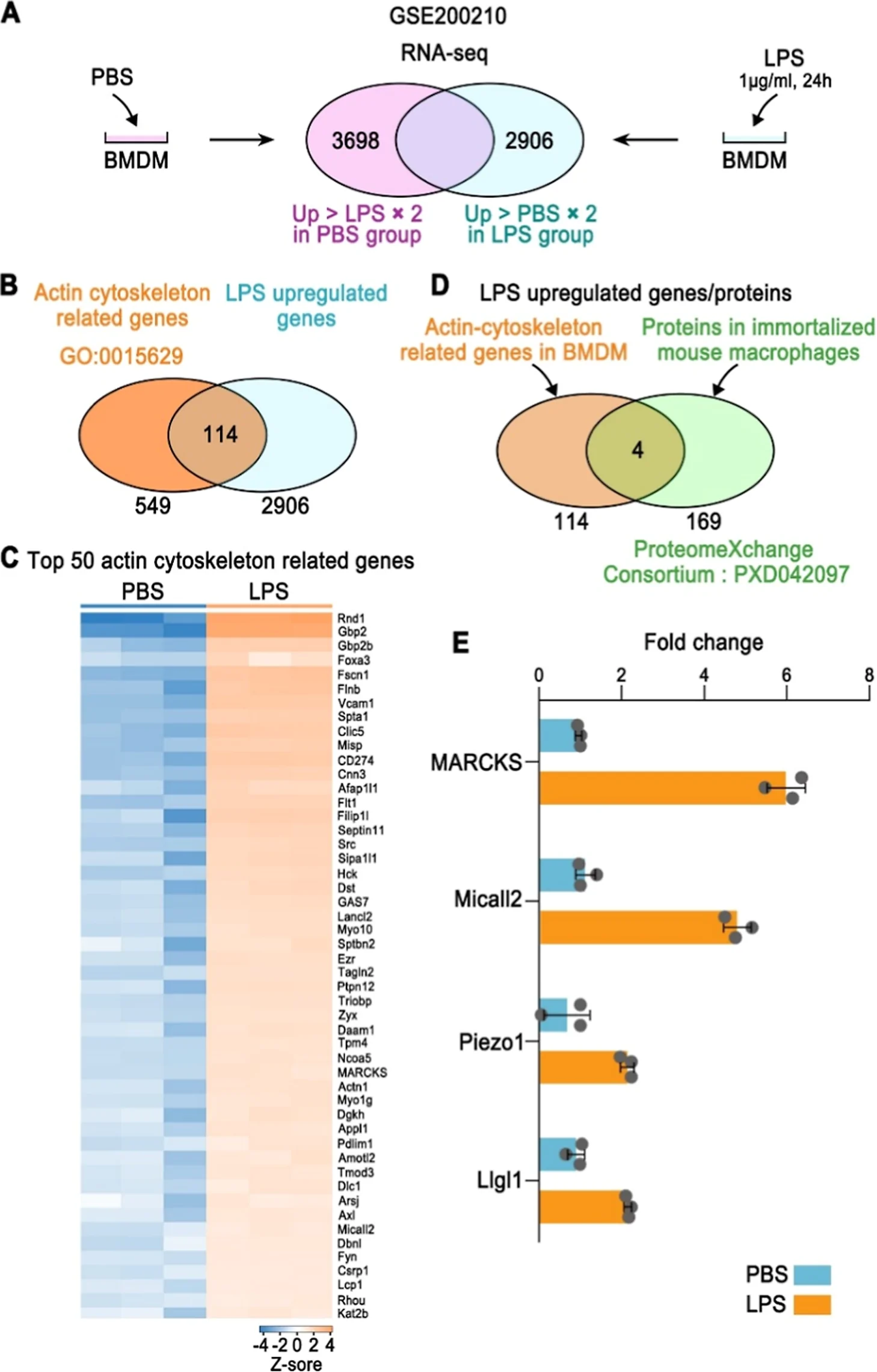
Actin cytoskeleton-related genes upregulated in macrophages following LPS treatment. (A) Schematic illustrating the gene selection process from RNA-seq, showing a 2-fold upregulation in LPS-treated BMDMs (cyan) compared to PBS-treated controls (magenta), identifying 2906 upregulated genes. (B) 114 upregulated actin cytoskeleton-related genes were selected based on comparison with an actin cytoskeleton gene database. (C) Heatmap of the top 50 actin cytoskeleton-related genes from panel B. (D) Four commonly upregulated genes identified by cross-referencing RNA-seq data with proteomic data. (E) Fold changes of the four selected genes (D) following LPS treatment, calculated from panel A data.

**Figure 4:**
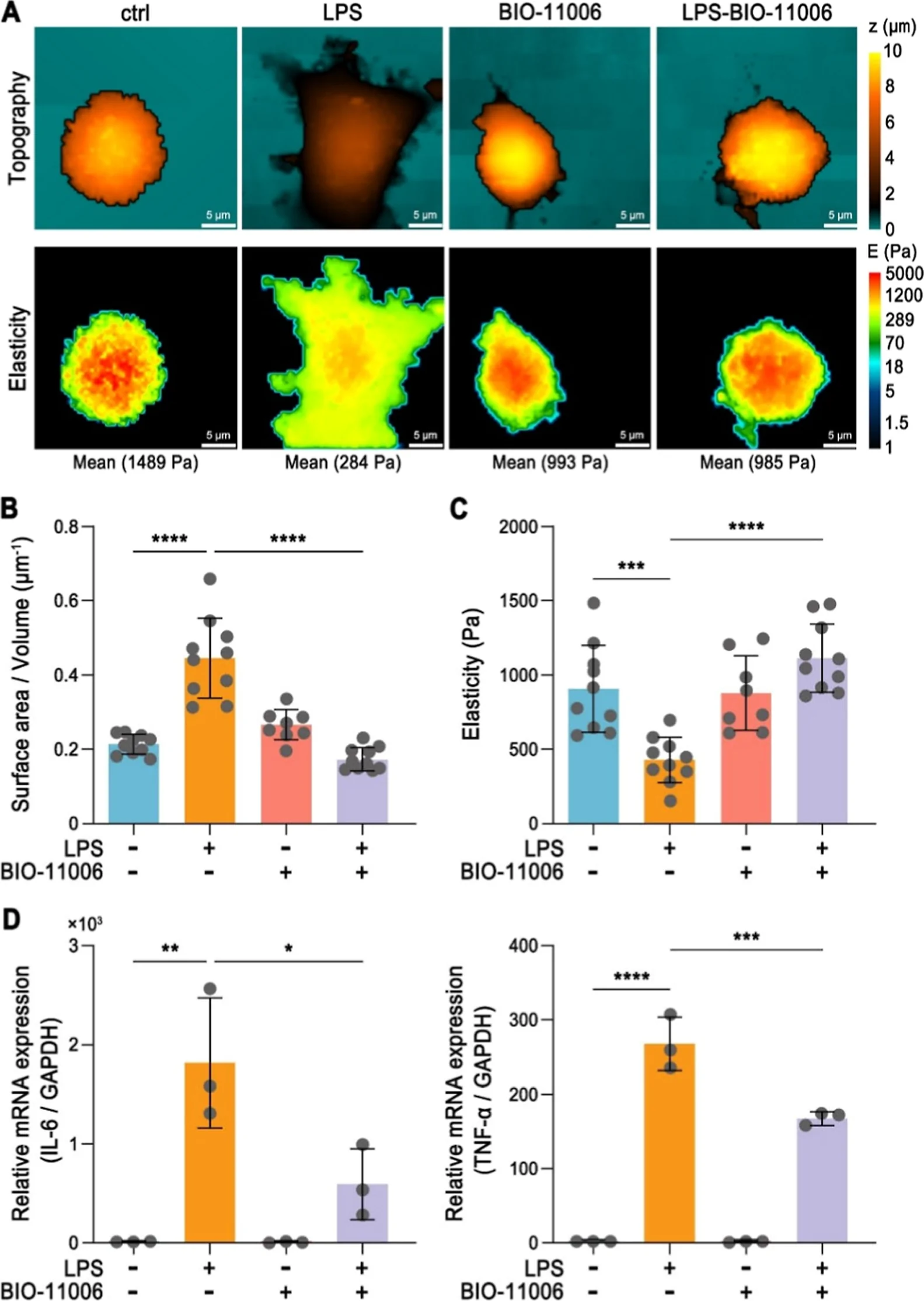
Inhibition of LPS-induced morphological, nanomechanical, and immune changes by MARCKS inhibitor (BIO-11006). (A) Representative SICM images of topography (upper) and elasticity (lower) of iBMDM cells under different conditions. Elasticity values are shown below each image, with height and elasticity color-coded as indicated by the scales on the right. Bars = 5 μm. (B) SA/V ratio of iBMDMs with different treatments. (C) Elasticity values of iBMDMs with different treatments. Each dot in B and C represents the value from an individual cell, pooled across three independent experimental repeats, *n* = 10 in each group. (D) mRNA expression levels of IL-6 (left) and TNF-α (right) in iBMDMs under various conditions. Data is from three independent experimental repeats. *p < 0.05, **p < 0.01, ***p < 0.001, ****p < 0.0001.

**Figure 5:**
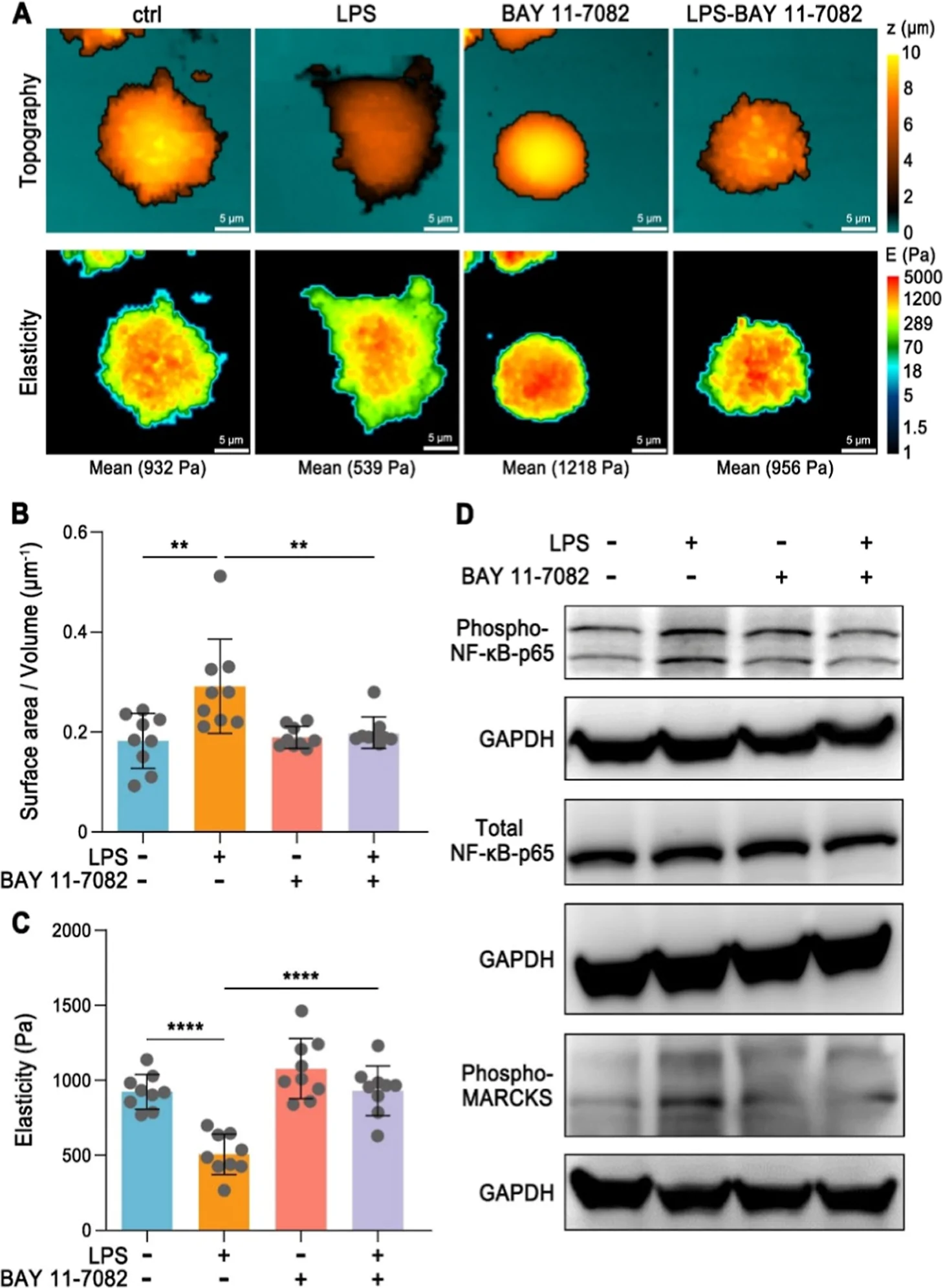
NF-κB-MARCKS axis regulates LPS-induced changes in elasticity, surface area-to-volume ratio (SA/V), and immune response. (A) Representative SICM images showing topography (top) and elasticity (bottom) of iBMDM cells under different treatments. Elasticity values are shown below each image. Height and elasticity are color-coded as indicated by the scales on the right. Bars, 5 μm. (B) SA/V ratio of iBMDM cells with different treatments. (C) Elasticity of iBMDM cells with different treatments. Each dot in B and C represents the value from an individual cell, pooled across three independent experimental repeats, *n* = 9 in each group. (D) Western blot analysis of phospho-NF-κΒ-p65, total NF-κΒ-p65, and phospho-MARCKS in iBMDM cells under different conditions. GAPDH was used as an internal control. *p < 0.05, **p < 0.01, ***p < 0.001, ****p < 0.0001.

## Results

### LPS Activation in Individual iBMDMs Results in Increased Cell Spreading, an Elevated Surface Area-to-Volume (SA/V) Ratio, and Decreased Cell Elasticity

Lipopolysaccharide (LPS) is a well-established activator of M1 macrophages through TLR4 signaling.^21^ To evaluate polarization, real-time PCR was performed on LPS-stimulated iBMDMs, using IL-6 and TNF-α as M1 markers and IL-10 and TGF-β1 as M2 markers.^30^ LPS significantly upregulated IL-6 and TNF-α without inducing IL-10 or TGF-β1 (Figure S1A), confirming M1 polarization. Western blot analysis further showed increased iNOS expression and activation of the NF-κB pathway, as indicated by elevated phosphorylation of p65 (Figure S1B). Together, these results confirm successful M1 activation under our experimental conditions.

Given the critical role of cellular morphology and mechanics in macrophage activation,^4,5^ we assessed 3D morphology, SA/V ratio, and elasticity of iBMDMs before and after LPS stimulation using SICM (Figures S2 and S3). M0 iBMDMs had a small, rounded morphology, while LPS-treated M1 macrophages became flattened and spread (Figure 1A). LPS significantly increased SA/V without changing cell volume, indicating substantial morphological remodeling (Figure S2). SICM mapping revealed that M1 iBMDMs were softer than M0 controls (Figure S4). As cellular stiffness is influenced by F-actin organization,^4,31^ we examined F-actin structure with phalloidin staining. In M0 cells, F-actin formed elongated, parallel stress fibers, consistent with higher stiffness.^22^ LPS treatment disrupted these fibers, leading to cytoskeletal reorganization with shorter F-actin bundles and overall softening (Figures S3 and S4).

To capture the real-time dynamics of LPS-induced M1 macrophage activation, we continuously monitored live iBMDMs for ∼2 h using repetitive SICM scanning of morphology and mechanical properties. Following LPS treatment, iBMDMs gradually exhibited morphological changes, characterized by spreading and an increased SA/V ratio, while untreated controls maintained a rounded shape with minimal changes (Figure 1B, C; Videos S1 and S2). Concurrently, LPS-treated cells progressively softened during M1 polarization, whereas M0 macrophages retained higher stiffness throughout (Figure 1B, D; Videos S1 and S2). These findings suggest that cell spreading, elevated SA/V, and reduced stiffness are key hallmarks of M1 activation and may enhance macrophage responses to pathogenic challenges.

### LPS-Induced F-Actin Remodeling and Its Role in iBMDM Morphology, Nanomechanical Softening, and Immune Response

F-actin cytoskeletal remodeling is known to regulate macrophage stiffness.^4,31^ To investigate whether F-actin dynamics contribute to LPS-induced changes in macrophage morphology, mechanics, and immune responses, iBMDMs were treated with Jasplakinolide (JAS), an F-actin polymerization inducer, to counteract LPS-induced softening. As expected, JAS treatment prevented LPS-induced cell spreading, SA/V elevation, and stiffness reduction, thereby suppressing both morphological remodeling and nanomechanical softening (Figure 2). Additionally, JAS significantly reduced IL-6 and TNF-α expression in LPS-treated iBMDMs, without affecting IL-10 or TGF-β1 levels (Figure 2D and Figure S5). These findings suggest that LPS-induced cellular softening, mediated by F-actin depolymerization, is critical for effective M1 macrophage activation.

### Upregulation of Actin Cytoskeleton-Related Gene, MARCKS, in LPS-Activated Macrophage

To identify the intracellular pathways regulating macrophage morphology and elasticity in response to LPS, we analyzed RNA sequencing data from LPS-treated BMDMs (GSE200210), revealing 2906 upregulated genes (Figure 3A). Cross-referencing with an actin cytoskeleton-related gene database, we identified 114 candidates (Figure 3B, C). Integrating these with proteomic data (PXD042097), we identified four consistently upregulated genes—MARCKS, Micall2, Piezo1, and Llgl1 (Figure 3D, E). Among these, MARCKS showed the highest upregulation (Figure 3E), aligning with its role in promoting IL-6 and TNF-α expression.^26^ These findings suggest that MARCKS mediates LPS signaling, influencing macrophage morphology, elasticity, and immune function. Activation of Marcks upon LPS stimulation in our experimental model further suggests its important role in regulating macrophage cell morphology and nanomechanics (Figure S6A).

### The NF-κB-MARCKS Signaling Axis Regulates Cellular Morphology and Nanomechanics in LPS-Activated Macrophages

MARCKS showed the most pronounced upregulation following LPS treatment (Figure 3E). Recent studies have demonstrated that LPS-induced phosphorylation of MARCKS promotes its translocation from the membrane to the cytoplasm, thereby facilitating actin reorganization and pro-inflammatory cytokine release.^32^ To investigate whether MARCKS mediates LPS-induced changes in SA/V and nanomechanical softening, iBMDMs were pretreated with the MARCKS inhibitor BIO-11006 for 30 min (Figure S6A) followed by LPS treatment and SICM analysis. BIO-11006 markedly attenuated LPS-induced cell spreading and the increase in SA/V (Figure 4A, B), confirming the involvement of MARCKS in inflammation-associated morphological remodeling. In parallel, BIO-11006 reversed the LPS-induced reduction in cell stiffness (Figure 4A, C), indicating that MARCKS contributes to macrophage nanomechanical softening during activation. Moreover, MARCKS inhibition selectively suppressed the expression of pro-inflammatory cytokines IL-6 and TNF-α in LPS-stimulated iBMDMs, while exerting minimal effects on anti-inflammatory cytokines IL-10 and TGF-β1 (Figure 4D and Figure S6B). Conversely, treatment with Phorbol 12-myristate 13-acetate (PMA), a MARCKS activator,^33,34^ robustly enhanced MARCKS activation (Figure S7A) and induced pronounced cell spreading together with a significant, dose-dependent decrease in cellular stiffness (100–500 nM; Figure S7B, C), closely mimicking the phenotype induced by LPS stimulation. Collectively, these findings identify MARCKS as a central regulator of macrophage morphological remodeling, nanomechanical properties, and pro-inflammatory responses during LPS-induced activation.

LPS-activated macrophages exhibit prolonged nuclear translocation of NF-κB in a dose-dependent manner.^35^ Given its central role in macrophage activation, we explored the relationship between MARCKS and NF-κB signaling. We used BAY 11-7082, an inhibitor blocking nuclear translocation of the p65 subunit,^36^ to confirm NF-κB’s involvement in LPS-induced morphological and nanomechanical changes. BAY 11-7082 reversed LPS-induced alterations in cell morphology (Figure 5A), SA/V ratio (Figure 5B), and elasticity (Figure 5C), and reduced IL-6 and TNF-α expression (Figure S8A), confirming NF-κB’s role in M1 polarization and macrophage nanomechanics. We then assessed if NF-κB regulates MARCKS activation and found that BAY 11-7082 significantly reduced LPS-induced MARCKS phosphorylation (Figure 5D), indicating NF-κB acts upstream of MARCKS. Finally, MARCKS inhibition by BIO-11006 displayed no effect on NF-κB activation (Figure S8B), suggesting MARCKS does not directly regulate NF-κB signaling. In conclusion, our results suggest that TLR4-induced NF-κB activation drives MARCKS phosphorylation, leading to macrophage membrane remodeling and nanomechanical softening.

## Discussion

Macrophages respond to immune challenges by rapidly adapting their mechanical and morphological properties, enabling migration, cytokine secretion, and pathogen clearance.^37–39^ Growing evidence in mechanobiology highlights that mechanical signals are as critical as biochemical cues in orchestrating immune activation.^4,40^ Consistent with this, we found that LPS stimulation—via TLR4-NF-κB signaling—induces pronounced cell spreading in iBMDMs, characterized by an elevated SA/V ratio, decreased stiffness, and increased pro-inflammatory cytokine production, including TNF-α and IL-6. While similar M1 phenotypes have been reported,^22,41,42^ our study provides the first quantitative measurement of dynamic SA/V changes in real time. This increase in surface area likely reflects the unfolding of intracellular membrane stores and exocytosis,^43,44^ enabling macrophages to expand their surface without increasing volume.^45,46^ These biophysical adaptations, driven by cytoskeletal remodeling, likely enhance macrophage responsiveness by increasing receptor density, phagocytic efficiency, and deformability during inflammation.^5^

While some studies have reported increased stiffness during LPS-induced M1 polarization in RAW264.7 and THP-1 macrophages,^10,12^ our time-resolved SICM nanomechanical mapping reveals a distinct, progressive softening during the transition from M0 to M1 polarization in individual macrophages. This finding aligns with AFM studies showing that softer M1 alveolar macrophages internalize SARS-coronavirus more efficiently^13^ and that LPS-stimulated human M1 macrophages exhibit reduced stiffness compared to resting M0 cells.^11^ Additionally, AFM-based analyses indicate that LPS decreases nuclear stiffness, potentially enhancing nuclear deformability and facilitating migration through dense stromal environments.^47^ In contrast, actin polymerization-induced stiffening in THP-1 macrophages has been shown to impair migration and lamellipodia formation.^48^ Environmental stiffness also influences macrophage phenotype: a soft ECM promotes M1 polarization with a spreading, round morphology, whereas a stiff ECM favors M2 polarization with an elongated shape.^7,8^ These results support the idea that macrophages dynamically adapt their stiffness to maintain mechanical homeostasis, consistent with our observation that M1 macrophages favor a softer state and a spreading morphology.

To regulate the observed morphological and nanomechanical changes during M1 polarization, our data show that LPS induces phosphorylation of the membrane-associated actin-binding protein MARCKS in iBMDMs. Pharmacological inhibition of MARCKS with BIO-11006 reverses LPS-induced increases in surface SA/V ratio and decreases in cellular stiffness, identifying MARCKS as a key regulator of inflammatory remodeling. Although MARCKS is highly expressed in macrophages and has been suggested to play a central role in cytoskeletal regulation and inflammation, its mechanisms of action have remained unclear.^25,26^ It is known to be upregulated by LPS through the TLR4-NF-κB pathway,^49,50^ and its phosphorylation promotes cytoplasmic translocation, actin reorganization, and morphological changes.^32^ MARCKS also contributes to directional migration by modulating cortical actin, a process critical for MCP-1-mediated migration and cytokine secretion in neutrophils.^32,51^

Our findings highlight the role of mechanobiology in M1 macrophage activation, demonstrating how nanomechanical and immunological signals integrate to drive inflammation. LPS-induced NF-κB activation triggers MARCKS phosphorylation and cytoplasmic translocation, initiating cytoskeletal remodeling, cell spreading, and softening, which in turn enhances pro-inflammatory cytokine expression. Under resting conditions, MARCKS stabilizes the actin cytoskeleton to support cell adhesion and motility.^32,52^ Upon activation via TLR4-NF-κB signaling, MARCKS phosphorylation weakens its membrane anchoring and promotes F-actin reorganization, thereby facilitating macrophage morphological remodeling and mechanical softening. Pharmacological inhibition of MARCKS (BIO-11006), NF-κB (BAY 11-7082), or F-actin stabilization with Jasplakinolide reversed LPS-induced softening and suppresses TNF-α and IL-6 expression in iBMDMs, supporting a functional role for MARCKS as a critical downstream effector of LPS-induced NF-κB signaling, linking inflammatory activation to macrophage morphology, nanomechanical properties, and functions.^47,51^ Consistently, PMA-mediated MARCKS activation induced macrophage spreading and cellular softening, phenocopying the effects of LPS stimulation (Figure S7). These findings support a central role for MARCKS in regulating macrophage morphological and nanomechanical remodeling during inflammatory activation. Mechanistically, MARCKS phosphorylation disrupts its membrane association and F-actin cross-linking activity, weakening cortical cytoskeletal integrity and promoting macrophage spreading and cellular softening.

Although the complete signaling cascade linking LPS stimulation to macrophage spreading and softening remains to be fully defined, our findings, together with existing literature,^29,33,50,51,53^ support a coordinated regulatory axis involving coordinated regulation by TLR4, NF-κB, MARCKS, and F-actin remodeling (Figure 6). In this framework, MARCKS emerges as a key mechanobiology regulator coupling inflammatory signaling to cytoskeletal dynamics, cellular mechanics, and immune activation, highlighting its potential as a therapeutic target for modulating early M1 pro-inflammatory responses.

**Figure 6:**
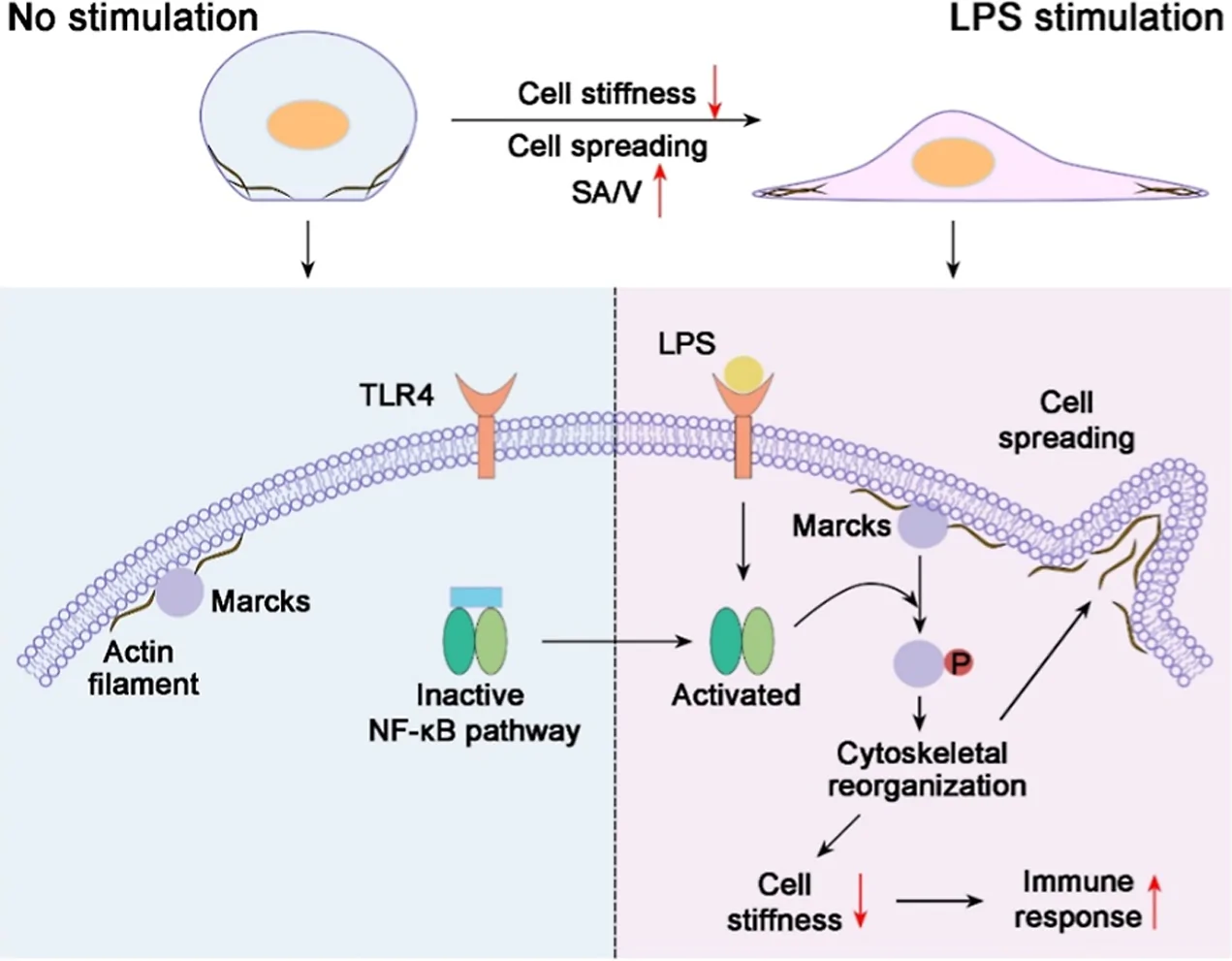
Schematic diagram illustrating the response mechanisms of macrophage during LPS-induced polarization.

## Conclusion

In this study, we used SICM to characterize the dynamic morphological and nanomechanical changes associated with LPS-induced macrophage polarization. We found that LPS stimulation increased the SA/V ratio and decreased cellular stiffness. Mechanistically, our data suggests that LPS activates the NF-κB signaling pathway, leading to MARCKS activation and subsequent cytoskeletal remodeling, which underlies the observed morphological transformation and mechanical softening of macrophages.

## Supplementary Material







## Notes

M1 and M2 macrophage markers, iNOS, and phospho-NF-κΒ-p65 analysis in LPS activated iBMDMs; LPS-activated iBMDMs show an increased surface area-to-volume ratio (SA/V) without a proportional change in volume; F-actin staining with Phalloidin in untreated and LPS-treated iBMDMs; LPS-treated iBMDMs show lower elasticity and a higher SA/V ratio than untreated control cells; Effects of F-actin enhancer jasplakinolide (JAS) on LPS-induced M2 markers expression in iBMDM cells; Effects of the MARCKS inhibitor BIO-11006 on LPS-induced MARCKS activation and M2 markers expression in iBMDM cells; Phorbol 12-myristate 13-acetate (PMA) induces MARCKS phosphorylation, which reduces cellular elasticity and promotes spreading of iBMDMs; Effects of the NF-κB inhibitor BAY 11-7082 on LPS-induced M1 and M2 markers, and of the MARCKS inhibitor BIO-11006 on NF-κB activation in iBMDMs; SEM image of SICM scanning nanopipette (PDF)
Morphological and mechanical dynamics of a single untreated (MP4)
Morphological and mechanical dynamics of a LPS-treated iBMDM (MP4)
