# Supplementary material for: Real-Time Scanning Ion Conductance Microscopy Reveals MARCKS-Driven Morphological Remodeling and Nanomechanical Softening during LPS-Induced Macrophage Polarization
Source: Anal Chem. 2026 Aug 5;98(32):23475–86. doi: 10.1021/acs.analchem.6c01115 (PMC13492044; doi:10.1021/acs.analchem.6c01115)
Supplement: Supplementary file 1 [file ac6c01115_si_001.pdf]

Supplementary Information

Real-time scanning ion conductance microscopy reveals MARCKS-driven morphological remodeling and nanomechanical softening during LPS-induced macrophage polarization

Dong Wang,[a] Ritsuko Yamase,[a] Kyunghee Park,[a] Hiromi Nishikawa,[a] Andrew Shevchuk,[a,b] Rebecca C. Coll,[c] Yuri Korchev,[a,b] Wei Wang,[*,c] Yanjun Zhang[*,a,b]

[a] WPI Nano Life Science Institute (WPI-NanoLSI), Kanazawa University, Kanazawa 920-1192, Japan

[b] Department of Medicine, Imperial College London, London W12 0NN, United Kingdom

[c] Wellcome-Wolfson Institute for Experimental Medicine, Queen's University Belfast, Belfast BT9 7BL, United Kingdom

[*]**Corresponding authors: wei.wang@qub.ac.uk, yanjunzhang@staff.kanazawa-u.ac.jp**

Table of contents:

Supplementary Figure S1 .... S2
Supplementary Figure S2 .... S3
Supplementary Figure S3 .... S4
Supplementary Figure S4 .... S5
Supplementary Figure S5 .... S6
Supplementary Figure S6 .... S7
Supplementary Figure S7 .... S8
Supplementary Figure S8 .... S9
Supplementary Figure S9 .... S10
Supplementary video information S11

Supplementary Figure S1

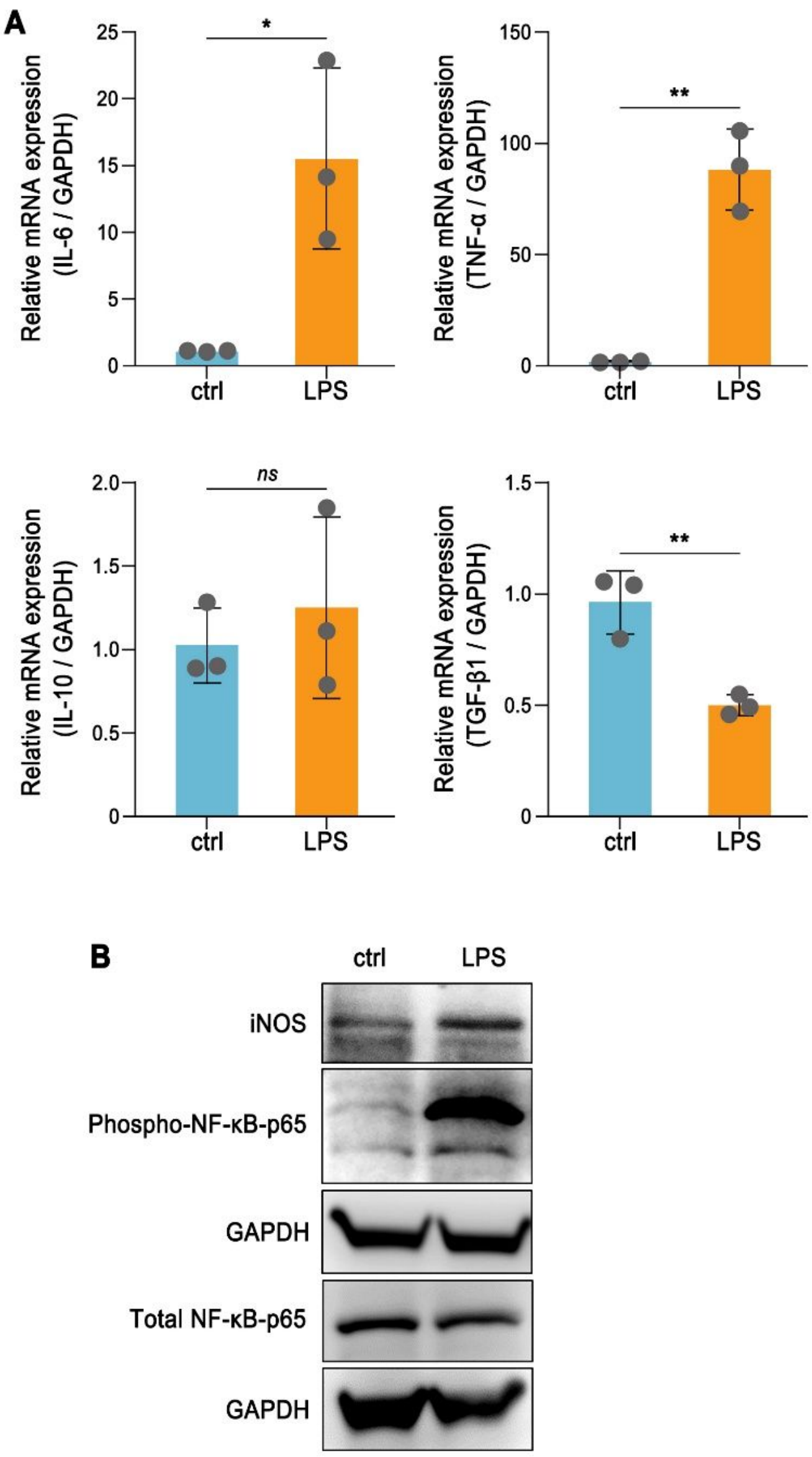

Supplementary Figure S1. LPS treatment activates iBMDMs. (A) M1 and M2 macrophage markers analysis after LPS treatment. Bar graph showing the relative mRNA expression levels of IL-6, TNF-α, IL-10, and TGF-β1 in untreated (ctrl) and LPS-treated iBMDM cells. Each dot represents the value from an individual experiment, pooled across three independent experimental repeats. *$p < 0.05$, ** $p < 0.01$, *** $p < 0.001$, **** $p < 0.0001$. *ns*, not significant. (B) Western blot analysis of iNOS, phospho-NF-κB-p65, and total NF-κB-p65 in LPS-treated iBMDM cells. GAPDH was used as an internal control.

Supplementary Figure S2

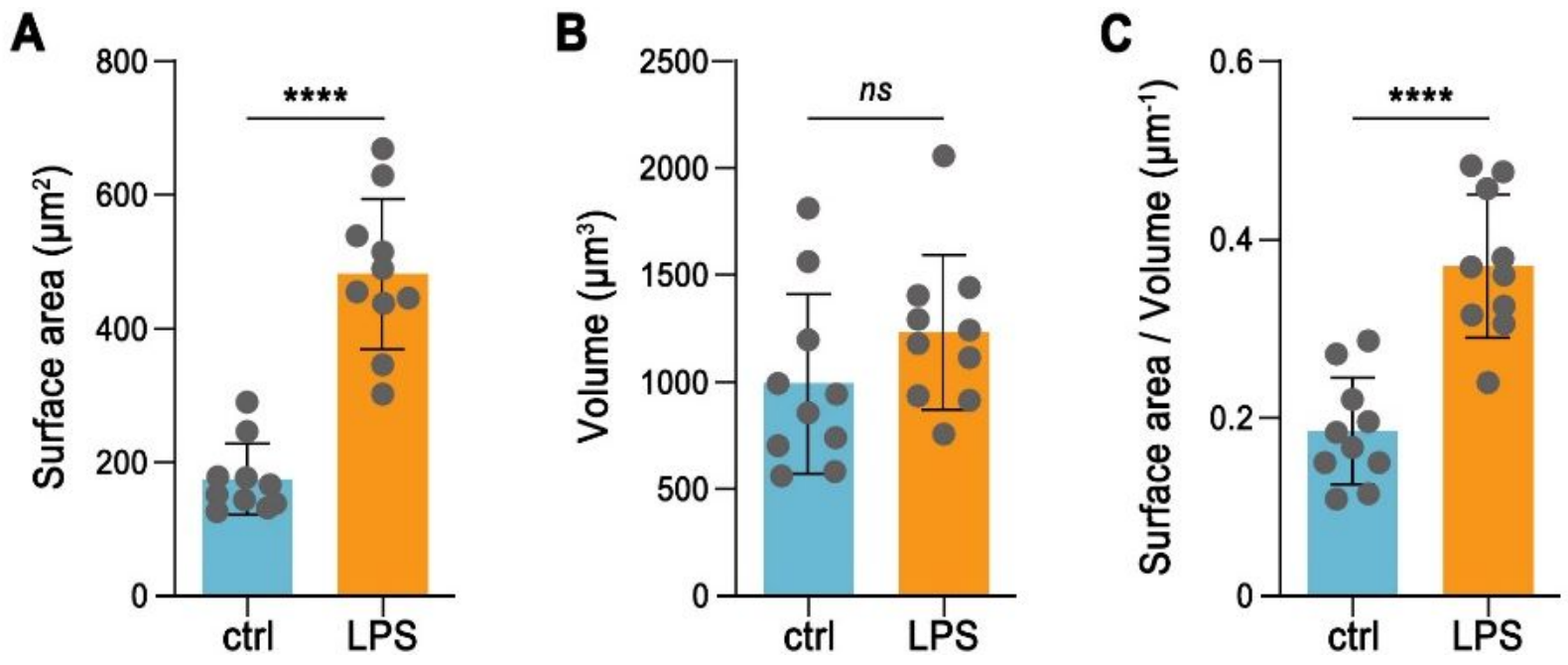

Supplementary Figure S2. LPS-activated iBMDMs show an increased surface area-to-volume ratio (SA/V) without a proportional change in volume. Bar graphs show the surface area (A), volume (B), and surface area-to-volume ratio (C) of untreated (ctrl) and LPS-treated iBMDM cells. Each dot represents the value from an individual cell, pooled across three independent experimental repeats, n=10 in each group. *p < 0.05, ** p < 0.01, *** p < 0.001, **** p < 0.0001. *ns*, not significant.

Supplementary Figure S3

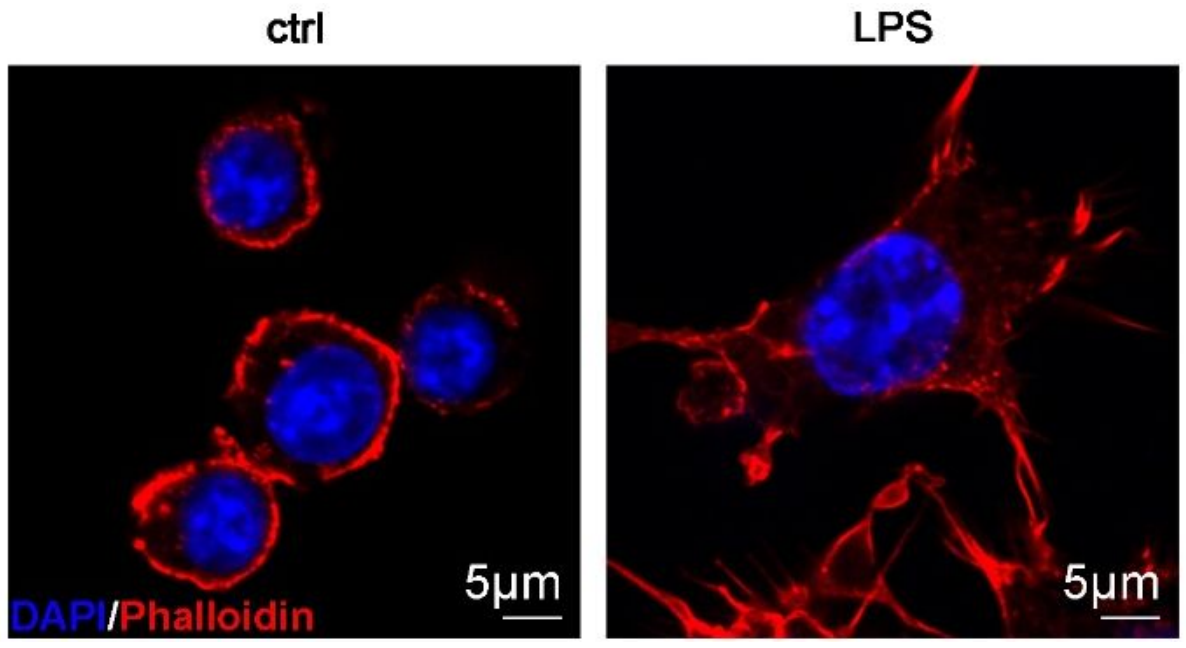

Supplementary Figure S3. F-actin staining with Phalloidin in untreated (ctrl) and LPS-treated iBMDMs. Bar = 5 μm.

Supplementary Figure S4

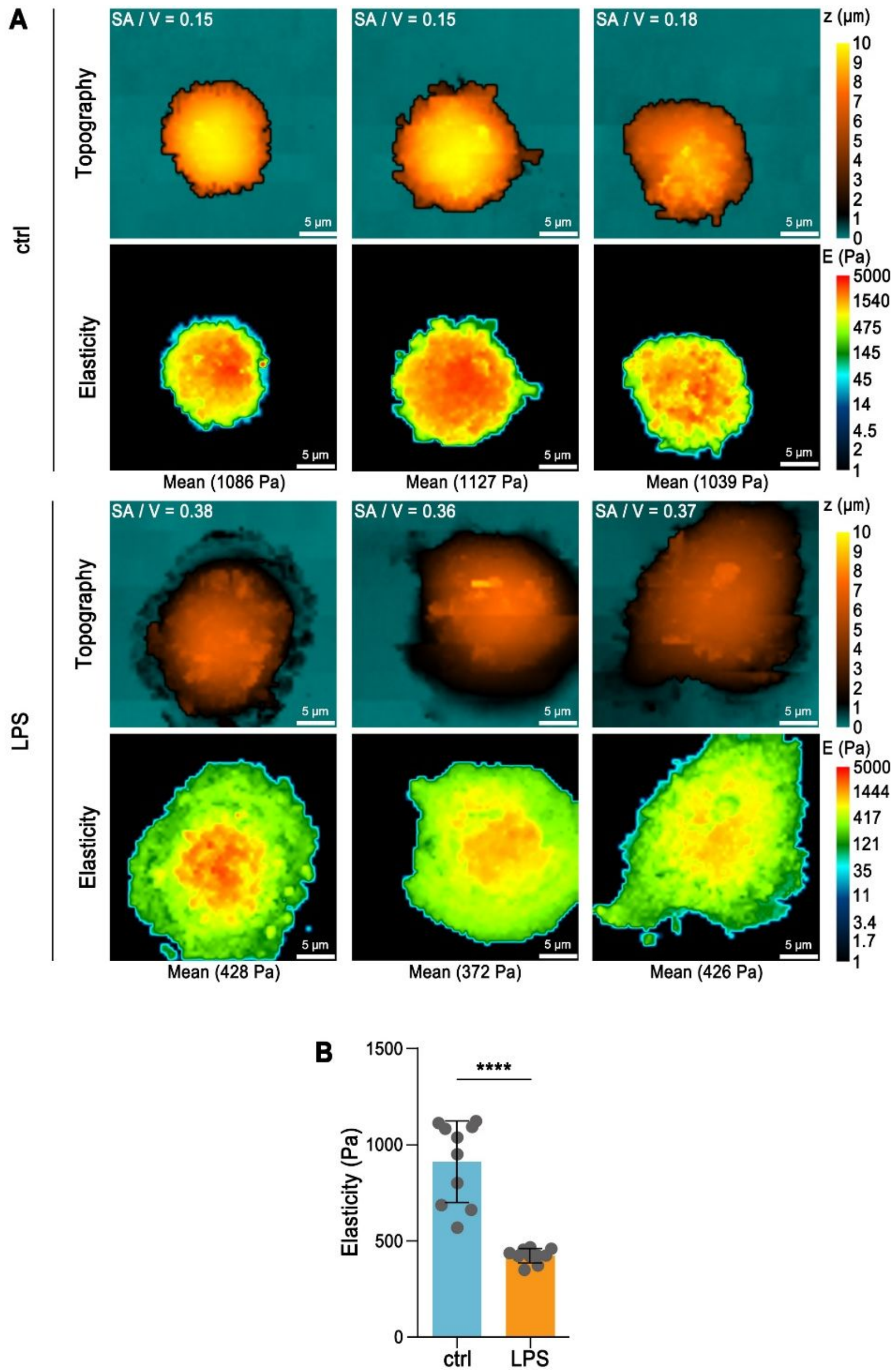

Supplementary Figure S4. LPS-treated iBMDMs show lower elasticity and a higher SA/V ratio than untreated control cells. (A) Representative topographical (upper panel) and elasticity-mapping (lower panel) images of untreated (ctrl) and LPS-treated iBMDM cells, acquired by SICM. Surface area-to-volume ratio (SA/V) and elasticity values are displayed in the images. Height and elasticity are color-coded as indicated on the right. Bar = 5 µm. (B) Bar graph showing elasticity values of untreated (ctrl) and LPS-treated iBMDM cells. Each dot represents the value from an individual cell, pooled across three independent experimental repeats, n=10 in each group. *p < 0.05, ** p < 0.01, *** p < 0.001, **** p < 0.0001. *ns*, not significant.

Supplementary Figure S5

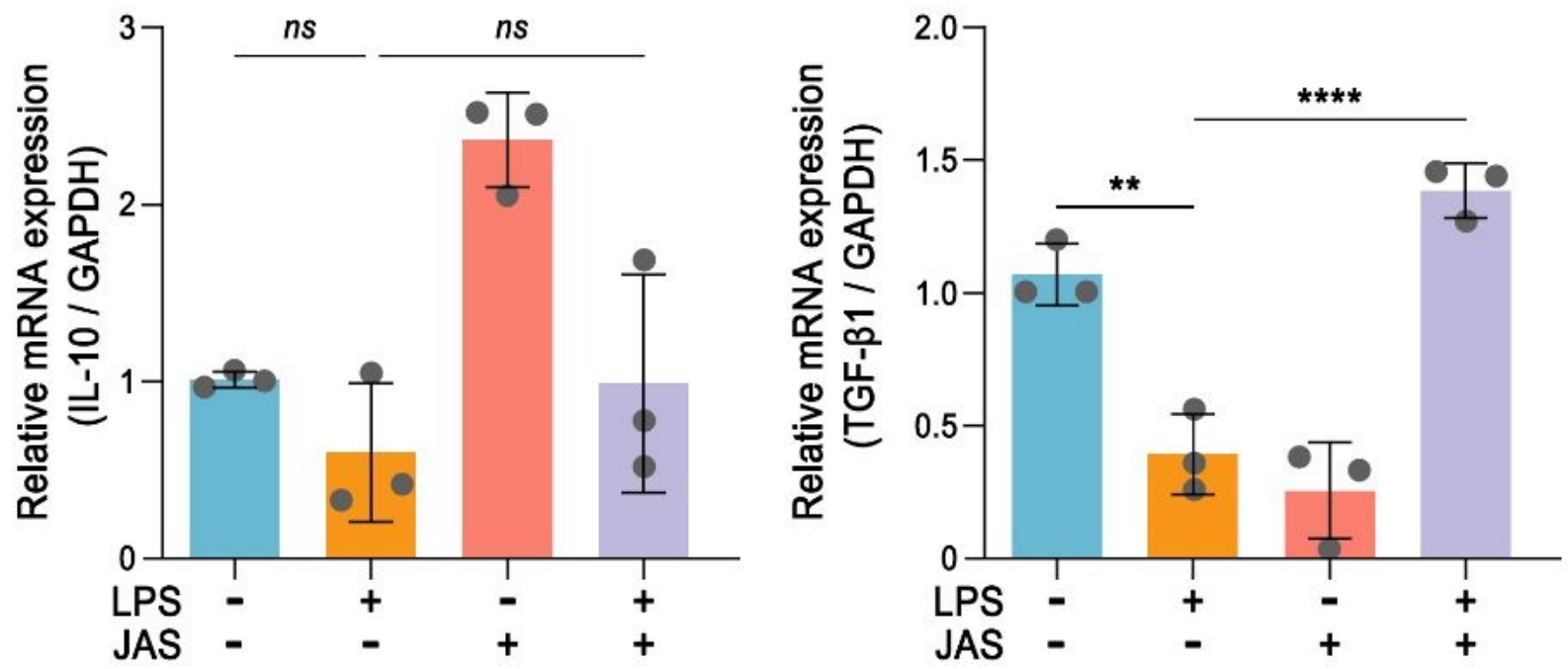

Supplementary Figure S5. Effects of F-actin enhancer jasplakinolide (JAS) on LPS-induced M2 markers expression in iBMDM cells. Bar graph shows the relative mRNA expression level of IL-10 and TGF-β1 in untreated and LPS-treated iBMDM cells. Each dot represents the value from an individual experiment, pooled across three independent experimental repeats. *$p < 0.05$, ** $p < 0.01$, *** $p < 0.001$, **** $p < 0.0001$. *ns*, not significant.

Supplementary Figure S6

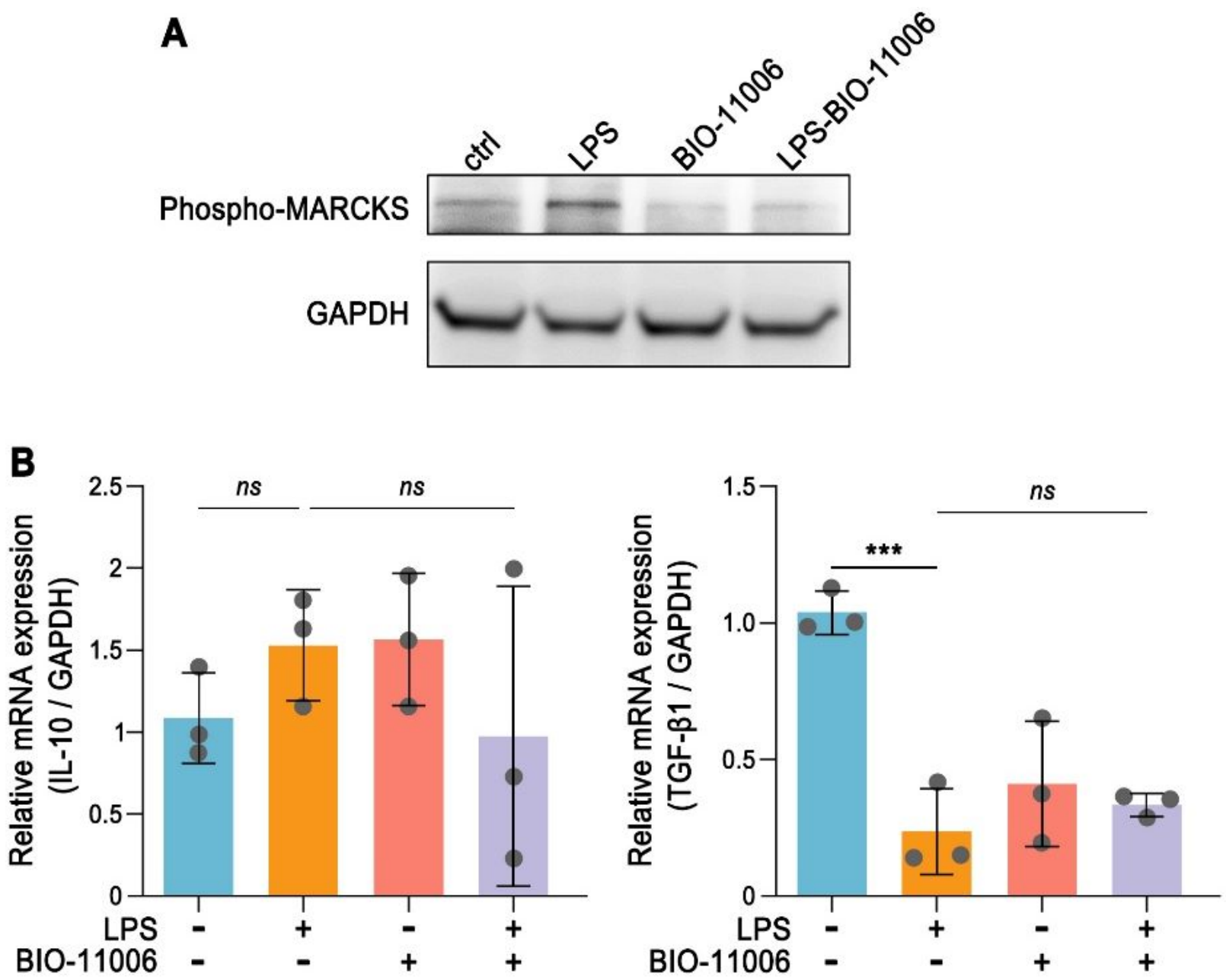

Supplementary Figure S6. Effects of the MARCKS inhibitor BIO-11006 on LPS-induced MARCKS activation and M2 markers expression in iBMDM cells. (A) Western blotting results of phospho-MARCKS in iBMDM cells with different treatments are shown. GAPDH was used as an internal control. (B) Bar graph shows the relative mRNA expression level of IL-10 and TGF-β1 in untreated and LPS-treated iBMDM cells. Each dot represents the value from an individual experiment, pooled across three independent experimental repeats. *$p < 0.05$, ** $p < 0.01$, *** $p < 0.001$, **** $p < 0.0001$. *ns*, not significant.

Supplementary Figure S7

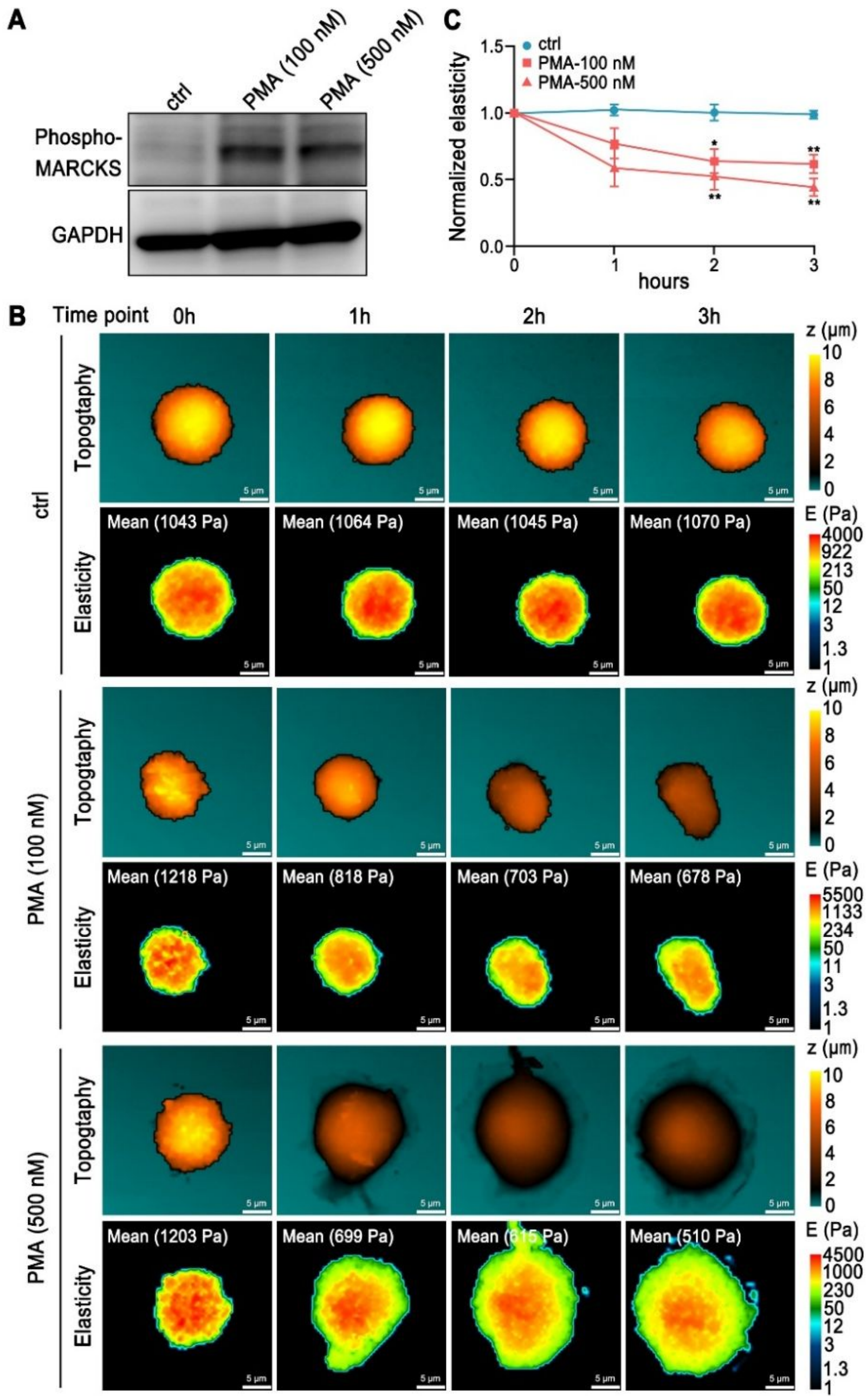

Supplementary Figure S7. Phorbol 12-myristate 13-acetate (PMA) induces MARCKS phosphorylation, which reduces cellular elasticity and promotes spreading of iBMDMs. (A) Western blotting results of phospho-MARCKS in control and PMA (100 nM and 500 nM) treated iBMDM cells are shown. GAPDH was used as an internal control. (B) Representative topographical (upper panel) and elasticity-mapping (lower panel) images of a continue SICM scanning of iBMDM cells before PMA treatment (ctrl) and after PMA-treatment over the marked time point, acquired by SICM. Height and elasticity are color-coded as indicated on the right. Bar = 5 μm. (C) Changes in elasticity of iBMDM cells over time under PMA treatment and control. Each dot represents the value from an individual cell, pooled across three independent experimental repeats. *p < 0.05, ** p < 0.01.

Supplementary Figure S8

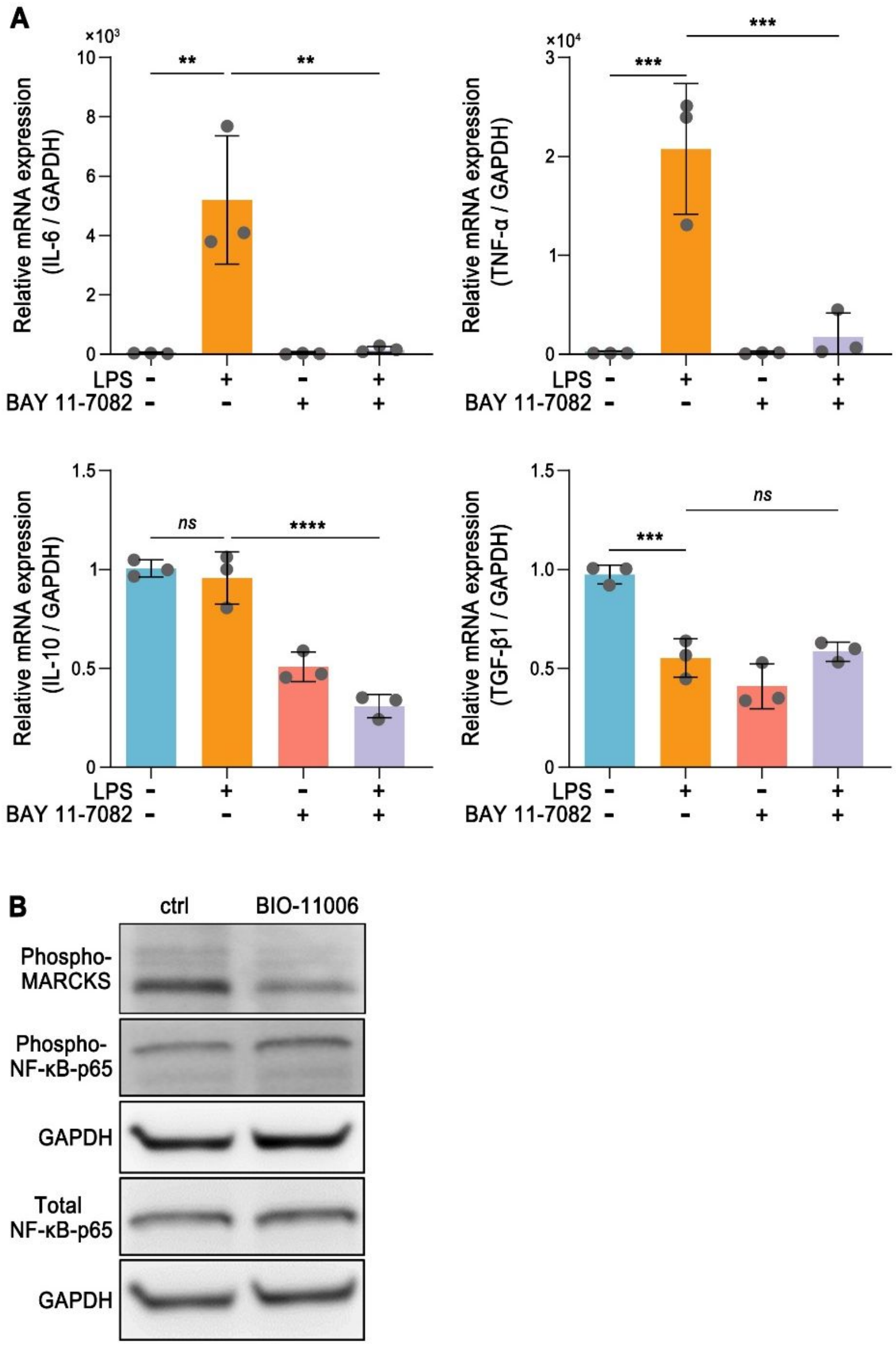

Supplementary Figure S8. Effects of the NF-κB inhibitor BAY 11-7082 on LPS-induced M1 and M2 markers, and of the MARCKS inhibitor BIO-11006 on NF-κB activation in iBMDMs. (A) Effects of the NF-κB inhibitor BAY 11-7082 on M1 and M2 markers in iBMDM cells. Bar graph shows the relative mRNA expression level of IL-6, TNF-α, IL-10 and TGF-β1. Each dot represents the value from an individual experiment, pooled across three independent experimental repeats. *$p < 0.05$, ** $p < 0.01$, *** $p < 0.001$, **** $p < 0.0001$. *ns*, not significant. (B) Effects of the MARCKS inhibitor BIO-11006 on NF-κB activation in iBMDM cells. Western blotting results of phospho-MARCKS, phospho-NF-κB-p65 and total NF-κB-p65 in control and Bio11006 treated iBMDM cells are shown. GAPDH was used as an internal control.

Supplementary Figure S9

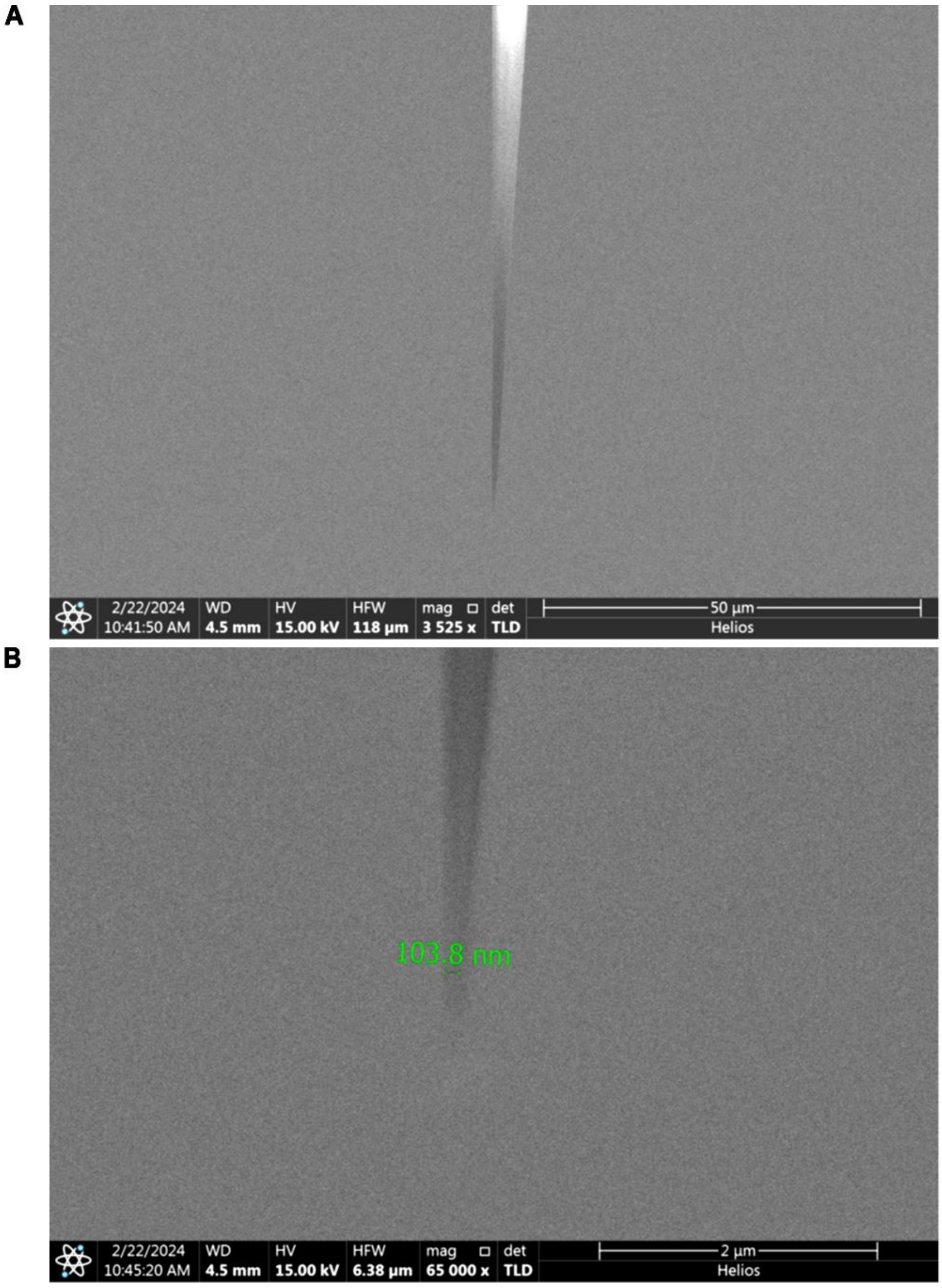

Supplementary Figure S9. SEM image of SICM scanning nanopipette. (A) Low-magnification view showing the overall morphology and geometry of the nanopipette. (B) Higher-magnification image of the tip region in (A), revealing a tip diameter of approximately 100 nm and a half-cone angle α of about 3°.

Supplementary Video 1. Morphological and mechanical dynamics of an untreated single iBMDM.

Supplementary Video 2. Morphological and mechanical dynamics of a single LPS-treated iBMDM.
